# Characterizing Social Determinants of Health in GI Cancer Surgery: Insights From the All of Us Research Program

**DOI:** 10.1002/cnr2.70518

**Published:** 2026-03-19

**Authors:** Manar Z. Al Rubaye, Kaleem S. Ahmed, Sheriff M. Issaka, Muhammad Maisam Ali, Benjamin A. Cher, Anas H. Awan, Nicci Owusu‐Brackett, Sharon M. Weber, Syed Nabeel Zafar

**Affiliations:** ^1^ Division of Surgical Oncology, Department of Surgery University of Wisconsin School of Medicine and Public Health Madison Wisconsin USA; ^2^ UCLA Henry Samueli School of Engineering and Applied Science Los Angeles California USA; ^3^ University of Nebraska Medical Center Omaha Nebraska USA; ^4^ University of Wisconsin Carbone Cancer Center Madison Wisconsin USA

## Abstract

**Introduction:**

Social Determinants of Health (SDoH) are increasingly recognized as critical contributors to cancer outcomes. Understanding SDoH among patients undergoing cancer surgery may reveal disparities that influence care and recovery. This study assessed the prevalence and variation of SDoH among patients undergoing gastrointestinal (GI) cancer surgery using a national dataset.

**Methods:**

We conducted a cross‐sectional analysis using the National Institutes of Health *All of Us* Research Program. Adults with GI cancer were identified using diagnosis codes, and surgery was confirmed using procedure codes. Participants who completed the SDoH survey were included. Descriptive statistics summarize demographics and SDoH domains: economic stability, healthcare access, neighborhood environment, and social context.

**Results:**

Of 6620 participants with GI cancer, 1747 underwent surgery; 470 (26.9%) completed the SDoH survey. Mean age was 69.4 years; 82.1% were White and 61.3% had college degrees. Most had health insurance (96.6%) and stable housing (88.7%), though 25.5% reported poor housing quality and 7.6% reported food insecurity. PROMIS T‐scores for physical and mental health were below average at 37.0 and 38.4, respectively.

**Conclusion:**

Despite favorable SDoH profiles, GI cancer surgery patients reported below average physical and mental health. These findings highlight the need to integrate multidomain SDoH data in cancer care research.

## 1 Introduction

Gastrointestinal (GI) cancers account for nearly 20% of all new cancer diagnoses and 28% of cancer‐related deaths in the United States [1]. Surgical intervention remains a cornerstone of curative treatment for many GI malignancies and plays a critical role in disease management across cancer types [2]. These operations are often complex, require significant healthcare resources, and involve multidisciplinary coordination and longitudinal care. Surgical patients frequently face extended recovery periods, heightened risks of complications, and significant logistical challenges related to perioperative care [2]. Both short‐ and long‐term surgical outcomes are influenced by a range of clinical factors, including age, disease stage, tumor grade, and functional status. However, growing evidence highlights the critical role of social determinants of health (SDoH) in shaping surgical outcomes, including risks of complications, treatment delays, and survival disparities [3, 4, 5].

SDoH are categorized into five domains including economic stability, education access and stability, neighborhood and built environment, social and community context, and healthcare access, and are increasingly recognized as key drivers of surgical outcomes [6, 7]. In the context of surgery, these factors have been associated with disparities in access to care, increased rates of postoperative complications, hospital readmissions, and long‐term recovery [3, 4, 5]. Within GI cancers specifically, prior studies have demonstrated that lower income and Medicaid or uninsured status are associated with advanced stage at presentation and worse survival for colorectal and pancreatic cancers [8, 9]. Neighborhood deprivation and low educational attainment have been linked to higher rates of delayed surgery and postoperative morbidity among patients undergoing colectomy and hepatectomy [10, 11]. Additionally, limited social support and transportation barriers have been shown to reduce adherence to multimodal therapy and follow‐up after GI cancer surgery [12, 13]. These findings underscore that social and structural factors are tightly intertwined with oncologic surgical outcomes in this population.

Although numerous studies have explored the impact of SDoH in cancer broadly, one critical gap remains. While the individual survey instruments that assess SDoH domains, such as the MOS Social Support Survey, the Perceived Stress Scale, and the Hunger Vital Sign, are well‐validated and have been used extensively in prior research, they have not previously been integrated within a single, nationally representative database that also links to electronic health records. The *All of Us* Research Program uniquely combines these validated tools with additional survey modules capturing less commonly studied constructs, such as healthcare utilization and neighborhood perceptions, allowing for a multidomain assessment of SDoH within one platform.

To address these gaps, we analyzed data from the National Institutes of Health All of Us Research program, a diverse national cohort that includes detailed survey‐based SDoH measures [14]. This study aims to characterize the prevalence and variation of SDoH among patients undergoing GI cancer surgery. This description will aid in the development of future hypotheses, research questions, and study design.

## 2 Methods

This study utilized de‐identified, cross‐sectional data from the National Institute of Health *All of Us* Research Program [14]. This comprehensive and diverse dataset includes electronic health records, biological specimens, physical examinations, and survey responses from over 560 000 participants, including individuals with cancer. The *All of Us* Research Program was specifically designed to build one of the most inclusive health databases in the United States, with the goal of enrolling at least 50% of participants from racial and ethnic minority or historically underrepresented populations. Its community‐based recruitment model intentionally targets participants from a wide range of geographic, socioeconomic, and clinical backgrounds, enabling analyses that capture the social and structural determinants of health.

Participants enroll either through the program's public website (direct volunteers) or via participating health care institutions, including academic centers and community organizations across the United States. Approximately two‐thirds of participants have enrolled through partner institutions and one‐third as direct volunteers, reflecting the program's dual strategy for broad representation. Participants enrolled through partner institutions can authorize automatic transfer of their EHRs to the *All of Us* database through secure data linkages. In contrast, direct volunteers without an affiliated institution may be unable to automatically link EHR data; for these individuals, only self‐reported survey data are available. Consequently, EHR completeness varies by enrollment pathway, which may introduce selection bias favoring participants receiving care at large, integrated health systems. However, inclusion of direct volunteers enhances demographic and geographic diversity by capturing participants who might otherwise be excluded from traditional institutional cohorts.

As this study involved secondary analysis of de‐identified data from the NIH All of Us Research Program, it was considered exempt from human subjects review under the U.S. Department of Health and Human Services regulations for exempt research (45 CFR §46.104).

All participants in All of Us provided informed consent to participate in the program, including permission for their data to be used for future research; this analysis used only de‐identified data accessed through the All of Us Researcher Workbench.

### 2.1 Patient Selection

We included all adult patients diagnosed with GI cancers who underwent a corresponding extirpative surgery and had available SDoH data in the dataset. Participants were identified using a combination of Systematized Nomenclature of Medicine (SNOMED) codes and International Classification of Diseases, Tenth Revision (ICD‐10) codes, specifically targeting primary and secondary malignant neoplasms across various regions of the GI tract. Both SNOMED and ICD‐10 codes are available in the *All of Us* dataset and were used together to ensure comprehensive case capture and minimize the risk of missing eligible participants, as some diagnoses are documented under one or both coding systems. SNOMED codes enabled identification of primary malignancies by specific GI region. ICD‐10 codes C15 through C26 were systematically applied to capture a comprehensive range of primary malignancies across GI regions. Secondary GI malignancies were identified through ICD‐10 codes within the C78 series.

CPT codes were then used to identify GI surgical procedures, and patients were included in the analytic cohort if they had both a qualifying GI cancer diagnosis and a GI surgical procedure code. For the patients with secondary metastasis, the specific sites of metastasis and the type of surgery performed were characterized in descriptive analyses. CPT code selection was based on *All of Us* concept sets and prior surgical oncology literature, with two authors independently reviewing and confirming relevant extirpative procedures. Disagreements were settled by the senior author (SNZ). To define the analytic cohort, only patients with both a qualifying GI cancer diagnosis and a GI surgical procedure code (using CPT codes) were included.

Gastrointestinal malignancies were identified using SNOMED and ICD‐10 diagnostic codes encompassing esophageal, gastric, hepatobiliary, pancreatic, small bowel, colorectal, and anal cancers (Supplementary Tables S1, S2). Surgical procedures were defined using CPT and ICD‐10‐PCS procedure codes corresponding to oncologic resections of the gastrointestinal tract (Supplementary Table S3).

### 2.2 Survey Tools

The SDoH survey provides an in‐depth assessment of participants' social and environmental contexts known to influence health outcomes. All participants in the *All of Us* Research Program are required to complete the Basics survey before proceeding to additional surveys, including the SDoH survey. The Basics survey collects essential background information, including country of birth, racial and ethnic identity, gender identity, biological sex at birth, sexual orientation, and marital status. It also captures household composition, economic indicators such as employment status, annual income, housing status, as well as health insurance coverage and recent financial stress indicators related to housing stability. Additionally, the Basics survey assesses disability status, querying daily activity challenges related to hearing, vision, mobility, cognition, and self‐care.

All surveys within the *All of Us* Research Program are self‐administered electronically by participants, either at enrollment sites or through the participant portal. Surveys may be completed at the time of enrollment or later during follow‐up, resulting in variable timing relative to surgical events. Because survey completion may occur before or after a participant's procedure, this temporal variability represents an important feasibility consideration when interpreting SDoH data in relation to surgery.

Most participants in this cohort also completed the Lifestyle, Overall, Health, and Healthcare Access and Utilization surveys. These additional surveys enriched the understanding of social determinants by providing context on health behaviors, general health status, and access to healthcare resources. Survey instruments were drawn from previously validated tools when available, such as the MOS Social Support Survey, Perceived Stress Scale, and Hunger Vital Sign. A subset of items, such as those assessing healthcare utilization and neighborhood environment, was developed by the *All of Us* program and are not independently validated.

### 2.3 Dataset and Variables

The analysis of SDoH utilized standardized scoring tools for each domain, which included social and community determinants, neighborhood and built environment, economic stability, and lifestyle. Each tool is briefly described below, with detailed analytical methods provided in the supplementary section.

#### 2.3.1 Social and Community Variables

Social cohesion and support were assessed through questions related to neighborhood trust, shared values, and mutual assistance using the RAND Medical Outcomes Study (MOS) Social Support Survey [15, 16]. The survey tool measured emotional and instrumental support, with higher percentages indicating stronger support. Consistent with prior RAND MOS survey applications, participants were divided into tertiles based on their total support score distribution within the cohort (low, medium, and high support). Loneliness was measured through the UCLA Loneliness Scale [17], which categorized participants into four groups: low, moderate, high, and very high loneliness. Experiences of general and healthcare‐specific discrimination were measured using a Likert scale (1–6) [18, 19]. Mean scores captured frequency, with higher means reflecting greater perceived discrimination. Stress was measured using the Perceived Stress Scale (PSS‐10), classifying participants into low, moderate, or high stress categories [20]. The Daily Spiritual Experience Scale (DSES) captured spiritual perceptions, though the absence of comparative groups precluded thresholds [21].

#### 2.3.2 Neighborhood and Built Environment Variables

The analysis of neighborhood and built environment variables utilized a structured questionnaire to capture participants' perceptions of physical and social disorder in the neighborhood, walkability, and access to public amenities. Physical and social disorder was assessed using the Ross‐Mirowsky Perceived Neighborhood Disorder Scale [22]; participants rated the prevalence of issues like graffiti, noise, crime, and drug use on a 1–4 Likert scale. Neighborhood crime, walkability, and residential density were adapted from the Physical Activity Neighborhood Environment Scale (PANES) [23, 24]. Crime and walkability were assessed on a 1–4 Likert scale and measured using a mean score. Residual density measured the count of participants within housing types: detached single‐family housing, mix of single‐family and town houses, townhouses, and apartments or condominiums.

#### 2.3.3 Economic Stability

Economic stability was assessed through food and housing insecurity indicators and housing quality reports, with positive responses indicating economic vulnerability. Food insecurity was assessed using the Hunger Vital Sign, a two‐question survey [25]. For housing instability, the HealthBegins Social Screening survey was utilized, which measured the amount of moves within 12 months [26]. Participants who moved more than two times in a year screened positive for housing instability. Finally, housing quality was assessed using the health‐related social needs (HRSN) survey from the Center for Medicare and Medicaid Services (CMS) [27].

#### 2.3.4 Lifestyle Survey

The Lifestyle survey documented health behaviors, including physical activity, diet, tobacco and alcohol use, and sleep patterns. The Overall Health survey captures self‐reported health status, physical and mental health symptoms, and diagnoses, while the Healthcare Access and Utilization survey was used to describe participants' use of healthcare services, usual sources of care, and potential barriers to access, such as insurance limitations and affordability. Alcohol consumption was analyzed using the AUDIT‐C scale, where a score of 4 or more in men and 3 or more in women indicated at‐risk drinking [28].

Health literacy was assessed using a 1–4 ordinal response scale derived from self‐reported ability to obtain, process, and understand basic health information. To avoid overlapping categories and improve interpretability, literacy scores were grouped into three mutually exclusive levels: low (≤ 2.0), moderate (> 2.0 to < 3.0), or high (≥ 3.0). These cutoffs were determined based on distributional tertiles within the analytical cohort and align with prior population health literacy literature.

Detailed scoring and categorization methods for individual SDoH domains are provided in Supplementary Appendices S1–S4, and the full statistical analysis plan is provided in Supplementary Appendix S5.

### 2.4 Statistical Analysis

Descriptive statistics, including means, standard deviations, and tertile distributions, were calculated for each domain of social determinants of health (SDoH). Categorical variables were summarized as counts and percentages, and continuous variables as means with standard deviations or medians with interquartile ranges, as appropriate. Group comparisons between participants who completed the SDoH survey and those who did not were performed using chi‐square tests for categorical variables, Student's *t*‐tests for normally distributed continuous variables, and Wilcoxon rank‐sum tests for non‐normal distributions.

Missing data were described using variable specific denominators, and item‐level completeness was summarized by survey instruments. All analyses were performed using Python. Two‐sided *p*‐values < 0.05 were considered statistically significant.

This study was deemed exempt from IRB review as it utilized only de‐identified data.

## 3 Results

**FIGURE 1:**
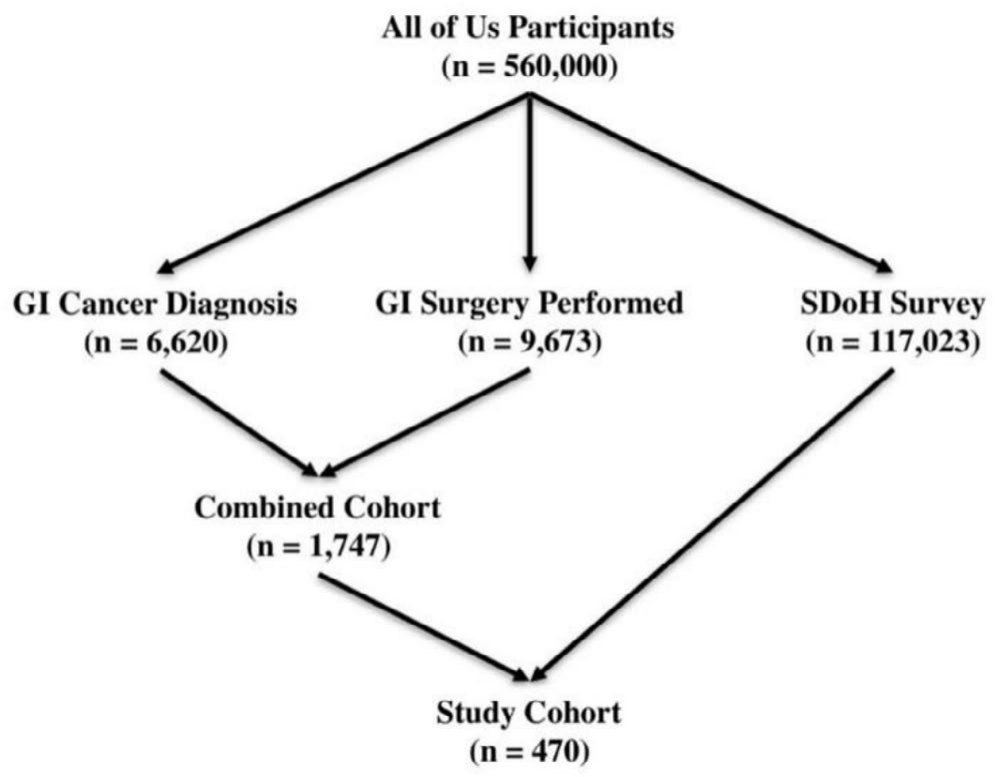
Selection of the final study cohort (*n* = 470) from the All of Us Research Program, including participants with a GI cancer diagnosis, GI surgery, and completed SDoH survey.

A total of 117 023 participants in the *All of Us* Research Program completed the Social Determinants of Health (SDoH) survey. Of these, 9673 participants were identified through this CPT‐based selection process as having undergone GI‐related procedures. To establish the final analytic cohort, only participants meeting both criteria: a confirmed GI cancer diagnosis (using ICD‐10 and SNOMED codes) and evidence of a GI surgical procedure (using the selected CPT codes). After applying both criteria, 1747 participants met inclusion for GI cancer diagnosis and surgery. Among these, 470 participants also completed the SDoH survey, forming the final study cohort. These individuals formed the study population, allowing for a focused analysis of SDoH factors among patients with documented GI cancer diagnoses who also underwent GI cancer surgery (Figure 1).

To assess potential selection bias, demographic characteristics of patients who completed the SDoH survey were compared with those who did not. This comparison was restricted to patients who met inclusion criteria for GI cancer diagnosis and surgery. Compared to patients who did not complete the SDoH survey, those who completed the survey were more likely to be older, White, female, and college educated (all *p* < 0.05). These findings highlight potential differences in demographic composition between survey responders and non‐responders (Table 1).

**TABLE 1:** Comparison of baseline characteristics between survey respondents and non‐respondents.

Characteristic	Completed SDoH survey (*n* = 470)	Did not complete SDoH survey (*n* = 1277)	*p*
Race: White	386 (82.1%)	695 (54.4%)	< 0.001
Sex: Female	245 (52.1%)	675 (52.9%)	0.828
College Graduate	288 (61.3%)	517 (40.5%)	< 0.001
Income ≥ $75 k	216 (46.0%)	316 (24.7%)	< 0.001
Insured	454 (96.6%)	1218 (95.4%)	0.328

The mean age was 69.4 years (SD = 11.5), with age categorized into 10‐year intervals for consistency with standard demographic reporting in cancer epidemiology. The largest proportion of participants was aged 71–80 years (188, 40.0%) (Table 2). Gender distribution was 240 (51.1%) female, 216 (46.0%) male, and 14 (3.0%) nonbinary or undisclosed. Most participants identified as straight (429, 91.3%), predominantly White (386, 82.1%), and non‐Hispanic (413, 87.9%). Educational attainment was high, with 288 (61.3%) reporting a college degree or more. Most participants were married or living with a partner (62.5%), followed by single (20.3%) and widowed or divorced (17.2%) (Table 2). The most common procedures were colectomies (189, 40.2%), followed by esophagectomies (43, 9.2%) (Table 2). Of the 470 patients, 50 (10.6%) had a diagnosis of secondary GI malignancy (ICD‐10 C78 series). The most common metastatic sites were retroperitoneum (*n* = 19), large intestine (*n* = 8), and liver (*n* = 7). Among these patients, colectomy (*n* = 24) and hepatectomy (*n* = 17) were the most frequently performed procedures, followed by enterectomy (*n* = 13) and cholecystectomy (*n* = 9).

**TABLE 2:** Demographics of study population (*n* = 470).

Variables	Categories	*N* (%)
Age Group, *n* (%)	18–50	35 (7.4)
	51–60	56 (11.9)
	61–70	109 (23.2)
	71–80	188 (40.0)
	81+	82 (17.4)
Gender Identity, *n* (%)^b^	Female	240 (51.1)
	Male	216 (46.0)
Sex Assigned at Birth, *n* (%)^b^	Female	245 (53.1)
	Male	216 (46.8)
Sexual Orientation, *n* (%)	Straight	429 (91.3)
	Non‐straight orientation, prefer not to answer, or skipped	41 (8.7)
Ethnicity, *n* (%)	Hispanic or Latino	33 (7.0)
	Not Hispanic or Latino	413 (87.9)
	PMI: Prefer Not To Answer, Skip, or None of these	24 (5.2)
Race, *n* (%)	White	386 (82.1)
	Black or African American	21 (4.5)
	Asian	*n* < 20^a^
	None indicated, none of these, prefer not to answer, skip, another single population, more than one population	60 (12.9)
Marital Status, *n* (%)^b^	Married	291 (61.9)
	Divorced/Separated/Widowed	111 (23.6)
	Never Married	42 (8.9)
	Living with partner	*n* < 20^a^
	Skip/prefer not to answer	*n* < 20^a^
Educational Attainment, *n* (%)^b^	College Graduate or advanced degree	288 (61.3)
	Highest Grade: College one to three years	125 (26.6)
	Highest Grade: Twelve or GED	39 (8.3)
	Less than a high school degree of equivalent	*n* < 20^a^
	Prefer not to answer/skip	*n* < 20^a^
Annual Household Income, *n* (%)	> 150 k	74 (15.7)
	100–150 k	71 (16.1)
	50–100 k	141 (30.0)
	< 50 k	109 (23.2)
	Prefer not to answer or skip	75 (16.0)
GI Cancer Surgery performed	Colectomy	189 (40.2)
	Esophagectomy	43 (9.2)
	Biliary tract	40 (8.5)
	Enterectomy	37 (7.9)
	Pancreatectomy	37 (7.9)
	Hepatectomy	36 (7.7)
	Gastrectomy	35 (7.5)
	Excision of rectal tumor	33 (7.0)
	Other	20 (4.3)

^a^ Cell counts fewer than 20 are suppressed in accordance with the All of Us Research Program's disclosure avoidance policy to protect participant confidentiality.

^b^ Missing data: sex assigned at birth, gender identity, marital status, and educational attainment.

Social Determinants of Health scores indicated moderate neighborhood support and cohesion, with an average score of 3.8 (SD = 0.4). The RAND MOS Social Support Survey showed a mean score of 3.9 (SD = 0.5), indicating moderate to high perceived social support (Table 3). Loneliness, measured using the UCLA Loneliness Scale, averaged 14.6 (SD = 4.8), suggesting low to moderate loneliness across the cohort. Daily Spiritual Experience scores averaged 3.7 (SD = 0.9) on a 0–6 scale, with higher scores indicating more frequent daily spiritual experiences, suggesting moderate levels of spiritual engagement. Perceived discrimination scores averaged 1.6 (SD = 0.5) on a 1–6 scale, with higher scores indicating greater frequency of perceived discrimination experiences. The Perceived Stress Scale revealed a mean stress score of 11.6 (SD = 7.0), indicating low to moderate stress levels among participants (Table 3). Cutoffs for these measures were based on established guidelines and tertile distributions derived from previous literature, ensuring a standardized interpretation of low, moderate, and high levels for each domain [20].

**TABLE 3:** Social and community construct measures.

Construct, (score range)	Mean (SD)	Median	Completed item (%)
Social Cohesion (1–5)	3.81 (0.48)	3.98	97.18
Social Support (1–5)	3.90 (0.52)	3.94	97.07
Loneliness (8–32)	14.61 (4.80)	14	97.53
Perceived Discrimination (1–6)	1.57 (0.54)	1.48	96.16
Perceived Stress (0–40)	11.63 (7.01)	11	94.5
Daily Spiritual Experience (0–6)	3.73 (0.87)	3.68	97.8

Table 4 summarizes neighborhood and built environment factors. The mean scores indicate moderate levels of physical (2.13, SD = 0.25) and social disorder (2.11, SD = 0.23), suggesting variability in participants' neighborhood conditions. Walkability received a slightly higher mean score (2.74, SD = 0.80), reflecting relatively favorable accessibility to physical activity‐promoting environments. However, crime levels were reported to be low, with a mean score of 1.40 (SD = 0.26), indicating that most participants reside in relatively safe neighborhoods.

**TABLE 4:** Neighborhood and built environment.

Construct	*N* items	Score range	Mean (SD)	Median	Item non‐responsive
Physical disorder	6	1–4	2.13 (0.25)	2.00	117 (4.2)
Social disorder	7	1–4	2.11 (0.23)	2.00	170 (5.2)
Walkability	5	1–4	2.74 (0.80)	2.00	97 (4.3)
Crime	2	1–4	1.40 (0.26)	1.00	112 (11.9)

Indicators of economic stability showed variability: 109 (23.2%) of participants **reported** an annual income below $50 000, and 141 (30.0%) reported between $50 000 and $100 000. Food insecurity was reported by 36 (7.6%) participants, who indicated that they sometimes or often lacked food in the past year. Housing stability was high, with 418 (88.7%) reporting no moves within the last 12 months. However, 120 (25.5%) reported housing quality issues, indicating concerns related to housing conditions.

Lifestyle factors included alcohol consumption, measured by the AUDIT‐C scale. Among men, 30 (14%) scored ≥ 4, and among women, 29 (12%) scored ≥ 3, meeting thresholds for at‐risk drinking. The mean AUDIT‐C score was **2.13 (SD = 1.89)** for men and **1.90 (SD = 1.86)** for women. Smoking history showed that 203 (43.1%) had smoked ≥ 100 cigarettes in their lifetime. Data on illicit drug use were not included in the analysis due to low response rates.

Regarding healthcare access, 454 (96.6%) participants reported having health insurance, with Medicare (257 [54.7%]) and employer‐provided insurance (177 [37.7%]) being the most common forms of coverage. Despite this, 18 (3.9%) reported delaying medical care due to cost, 29 (6.2%) reported affordability barriers for specialist visits, and 36 (7.7%) for prescription medications. Medical literacy was assessed on a 1–4 scale and demonstrated generally high levels across the cohort. Low medical literacy (score 1–2) was observed in 26 (6%) participants, moderate literacy (score > 2–< 3) in 79 (19%), and high literacy (score ≥ 3) in 320 (75%).

Health‐related quality of life was assessed using the PROMIS Global Health scales. Physical health scores averaged **37.02** (**SD = 1.90**) and mental health scores averaged **38.35** (**SD = 1.86**), both below the U.S. general population mean of 50, indicating below‐average physical and mental health among participants (Table 5).

**TABLE 5:** PROMIS (Patient‐Reported Outcomes Measurement Information System) for GI cancer participants.

	Global physical health	Global mental health
Mean, (SD)	37.02 (1.90)	38.35 (1.86)
Below 30	0 (0.00)	0 (0.00)
30–40	442 (0.94)	331 (0.0.70)
40–50	28 (0.06)	139 (0.30)
Above 50	0 (0.00)	0 (0.00)
Total	470 (1.00)	470 (1.00)

## 4 Discussion

This study reports the prevalence and variation of social determinants of health (SDoH) among patients undergoing gastrointestinal cancer surgery in the *All of Us* Research Program. Despite the cohort appearing socioeconomically advantaged, with most participants identifying as non‐Hispanic White, holding a college degree, and reporting favorable economic stability, neighborhood conditions, and insurance coverage, PROMIS assessments revealed lower‐than‐expected T‐scores for global mental and physical health [29]. This observation is descriptive and does not imply causality or lack of protective effects. These findings suggest a high burden of distress and poor self‐reported health among GI cancer surgery patients, even in the absence of overt socioeconomic disadvantage. This population faces a higher rate of complications and readmissions as they undergo complex, high‐risk procedures with prolonged recovery periods and chronic fear of recurrence following GI malignancy. These procedures are often preceded by delayed presentation, leading to diagnostic complexity and reinforcing the influence of social determinants of health on healthcare‐seeking behavior and access to timely diagnostic workup. Postoperatively, patients frequently experience delayed return of bowel function, dependence on enteral feeding, and long‐term effects of chemotherapy functional outcomes. These burdens, combined with social stressors, may contribute to the lower PROMIS scores observed. While prior literature has established that GI cancer surgery patients experience significant morbidity, there remains a need for large, representative studies linking social determinants and clinical outcomes, using datasets like *All of Us*, to generate novel insights across diverse populations and inform further interventions.

While long‐term survival among GI cancer patients has improved substantially over recent decades [1, 2], prior studies have shown persistent disparities by SDoH. For example, Singh et al. demonstrated that individuals from more deprived communities had higher cancer incidence and mortality [8], while Pinheiro et al. found that the presence of even one adverse SDoH was associated with elevated cancer mortality over a 10‐year follow‐up [9]. Similarly, McDougall et al. and other population‐based studies have shown that socioeconomic factors such as income, insurance status, and health literacy are associated with health‐related quality of life among cancer survivors [10, 30]. In contrast, our descriptive analysis identified lower perceived health despite generally favorable SDoH characteristics, suggesting that social advantage alone may not offset the psychological and social impact of complex cancer surgery. However, this study does not establish causal or protective relationships, as no comparative modeling or adjustment for confounders was performed. The observed pattern instead underscores the multifactorial nature of well‐being among surgical cancer patients.

Given these complexities, the findings reinforce the need for multidisciplinary, patient‐centered cancer care that accounts for both clinical and social determinants of health. While All of Us data cannot yet assess the impact of such interventions, future linkage studies could evaluate how integrated care models, including navigation, psychosocial support, and financial counseling, modify outcomes once longitudinal data are available [13, 31]. This analysis also highlights that the All of Us dataset enables multidomain assessment of SDoH but has practical limitations in representativeness and completeness. Compared to more traditional cancer registries such as SEER and the National Cancer Database (NCDB), the All of Us Research Program offers the advantage of patient‐level, survey‐based data across multiple validated domains [14, 32, 33]. However, the dataset is limited by voluntary participation, introducing self‐selection bias that likely over represents socioeconomically advantaged individuals [14, 34, 35]. Consistent with this, patients who completed the SDoH survey were more likely to be older, White, and college educated compared to non‐responders, highlighting potential selection bias.

Nonetheless, *All of Us* remains the only national‐level dataset that combines validated, multidomain SDoH instruments with individual‐level health and survey data [14]. One of the primary motivations for this study was to assess whether this resource can be used to support future research on the relationship between SDoH and cancer outcomes. Because outcomes data (e.g., complications, survival, recurrence) were not analyzed, this study cannot directly evaluate how SDoH influence cancer surgery outcomes. Instead, it provides descriptive insight into the distribution and completeness of SDoH measures within this population, which is informative in determining the design of future outcomes‐focused research.

Several limitations should be acknowledged. First, the sample may not be fully representative of the U.S. GI cancer surgery population, which limits generalizability [14, 32]. The distribution of racial and ethnic subgroups in the *All of Us* dataset does not reflect national demographics, likely due to its voluntary participation model, which introduces selection bias. Second, differences in institutional participation may contribute to referral bias, as the types of healthcare systems contributing data, and the populations they serve, are not evenly distributed geographically or demographically. Third, while *All of Us* provides extensive SDoH data, certain sensitive domains such as discrimination, financial strain, or illicit substance use may be underreported due to the self‐reported nature of the survey [18, 19, 27]. Fourth, cell counts below 20 are automatically suppressed in the public dataset to preserve privacy, which may obscure some sub‐group‐level variation. Fifth, the cross‐sectional nature of this analysis precludes causal inferences, making it difficult to determine whether specific SDoH factors directly contribute to poor mental or physical health. Notably, newer iterations of the *All of Us* program are working to address representativeness concerns by actively recruiting participants from historically underrepresented populations [14].

Another limitation is that the dataset does not allow for confirmation of whether the GI surgery was performed specifically for cancer or to determine surgical intent (curative or palliative). By including both primary and secondary malignancies, as well as all relevant GI surgeries, the cohort is more inclusive but also more heterogeneous, which may limit the specificity of conclusions regarding cancer‐directed procedures. Additionally, *All of Us* does not currently provide standardized clinical details on cancer staging, treatment modality, recurrence, or survival outcomes, which are routinely captured in SEER and NCDB [32, 33]. As a result, its current utility for longitudinal or outcomes‐based cancer research remains constrained. The number of GI cancer surgery patients with complete SDoH data also remains limited, with underrepresentation of individuals experiencing high social disadvantage. These factors reduce generalizability and should be considered in the design and interpretation of future studies.

Despite these limitations, this study provides a foundational step toward understanding the feasibility of using *All of Us* data to study SDoH among complex surgical cancer populations. Rather than inferring direct effects on outcomes, this analysis characterizes data completeness, demographic representativeness, and variability across SDoH domains. These are critical precursors to future analytic and interventional work. Future studies should evaluate how SDoH evolve throughout the cancer care continuum and how they interact with treatment access, recovery, and survivorship [11, 29]. Linking *All of Us* data with cancer registries or clinical sources could improve staging and treatment granularity, enabling more targeted and equitable interventions, particularly for patients undergoing complex surgery.

## 5 Conclusion

In summary, this study provides one of the first descriptive assessments of social determinants of health (SDoH) among patients undergoing gastrointestinal cancer surgery using the NIH *All of Us* Research Program. While many participants reported favorable socioeconomic conditions, including higher education, insurance coverage, and stable housing, self‐reported physical and mental health scores were below national averages, suggesting that the physiologic and psychological burdens of complex cancer surgery may persist despite socioeconomic advantage. Rather than evaluating outcomes, this analysis uses the *All of Us* data to characterize SDoH in surgical oncology populations and underscores the importance of integrating disease burden, treatment complexity, and recovery challenges in future research. With continued data expansion and linkage to cancer registries, the *All of Us* platform holds significant promise for advancing equity‐focused, multidomain investigations in oncologic surgery.

## Author Contributions

Manar Z. Al Rubaye led the study and participated in all components of the project, including study conceptualization and design; data acquisition, data management, and statistical analysis; interpretation of findings; comprehensive literature review; and drafting of the manuscript. Kaleem S. Ahmed, Sheriff M. Issaka, Muhammad Maisam Ali, Benjamin A. Cher, Anas H. Awan and Nicci Owusu‐Brackett contributed to data interpretation and critical review and feedback on manuscript drafts. Sharon M. Weber participated in study conceptualization, interpretation of results, and participated in critical review of the manuscript. Syed Nabeel Zafar provided senior mentorship, participated in all aspects of the study from conceptualization, data acquisition, analysis, interpretation of results, and manuscript writing. All authors reviewed and approved the final version of the manuscript.

## Funding

Dr. Syed Nabeel Zafar receives partial salary support for research from the NIH/NCI Early‐Stage Surgeon Scientist Program (Grant No. P30 CA014520‐48S4). No additional external funding was received for this study.

## Conflicts of Interest

The authors declare no conflicts of interest.

## Supporting information


**Table S1:** SNOMED Codes for Primary Malignant Neoplasms of the GI Tract.
**Table S2:** ICD‐10 Codes for Malignant Neoplasms of the GI Tract.
**Table S3:** CPT and ICD‐10‐PCS Codes for GI Cancer Surgery Procedures.
**Table S4:** Comparison of Baseline Characteristics between Survey Respondents and Non‐Respondents.
**Appendix S1:** Analytical Methods for Social and Community Context Measures.
**Appendix S2:** Analytical Methods for Neighborhood and Built Environment Measures.
**Appendix S3:** Analytical Methods for Economic Stability Measures.
**Appendix S4:** Analytical Methods for Lifestyle, Health Behaviors, and PROMIS Outcomes.
**Appendix S5:** Statistical Analysis Plan.

## Data Availability

The data that support the findings of this study are available through the NIH All of Us Research Program. Access to the data is subject to approval and compliance with programmatic and institutional requirements.
