# Supplementary material for: Characterizing Social Determinants of Health in GI Cancer Surgery: Insights From the All of Us Research Program
Source: Cancer Rep (Hoboken). 2026 Mar 19;9(3):e70518. doi: 10.1002/cnr2.70518 (PMC13093418; doi:10.1002/cnr2.70518)
Supplement: Supplementary file 1 — Table S1: SNOMED Codes for Primary Malignant Neoplasms of the GI Tract. Table S2: ICD‐10 Codes for Malignant Neoplasms of the GI Tract. Table S3: CPT and ICD‐10‐PCS Codes for GI Cancer Surgery Procedures. Table S4: Comparison of Baseline Characteristics between Survey Respondents and Non‐Respondents. Appendix S1: Analytical Methods for Social and Community Context Measures. Appendix S2: Analytical Methods for Neighborhood and Built Environment Measures. Appendix S3: Analytical Methods for Economic Stability Measures. Appendix S4: Analytical Methods for Lifestyle, Health Behaviors, and PROMIS Outcomes. Appendix S5: Statistical Analysis Plan. [file CNR2-9-e70518-s001.docx]

# **Supplementary Materials**

## **Table of Contents**

| **Section** | **Title** | **Page** |
| --- | --- | --- |
| Table S1 | SNOMED Codes for Primary Malignant Neoplasms of the GI Tract | 2 |
| Table S2 | ICD-10 Codes for Malignant Neoplasms of the GI Tract | 2–3 |
| Table S3 | CPT and ICD-10-PCS Codes for GI Cancer Surgery Procedures | 3–7 |
| Table S4 | Distribution of Secondary GI Malignancy Diagnoses and Corresponding Surgical Procedures | 8 |
| Appendix S1 | Analytical Methods for Social and Community Context Measures | 8–9 |
| Appendix S2 | Analytical Methods for Neighborhood and Built Environment Measures | 9 |
| Appendix S3 | Analytical Methods for Economic Stability Measures | 9–10 |
| Appendix S4 | Analytical Methods for Lifestyle, Health Behaviors, and PROMIS Outcomes | 10 |
| Appendix S5 | Statistical Analysis Plan | 10 |

**Method**

**GI Cancer Diagnosis Codes (SNOMED and ICD-10)**

**Table 1. SNOMED Codes for Primary Malignant Neoplasms of the GI Tract**

| **GI Cancer Type** | **SNOMED Code** |
| --- | --- |
| Primary malignant neoplasm of GI tract | 363745004 |
| Primary malignant neoplasm of Esophagus | 371984007 |
| Primary malignant neoplasm of Lower Third of Esophagus | 371998004 |
| Primary malignant neoplasm of Stomach | 372014001 |
| Primary malignant neoplasm of Duodenum | 93775003 |
| Primary malignant neoplasm of Small Intestine (Cecum) | 371977004 |
| Primary malignant neoplasm of Colon | 93761005 |
| Primary malignant neoplasm of Sigmoid Colon | 94006002 |
| Primary malignant neoplasm of Rectosigmoid Junction | 93980002 |
| Primary malignant neoplasm of Ascending Colon | 93683002 |
| Primary malignant neoplasm of Transverse Colon | 94105000 |
| Primary malignant neoplasm of Descending Colon | 93771007 |
| Primary malignant neoplasm of Splenic Flexure of Colon | 94072004 |
| Primary malignant neoplasm of Appendix | 93679002 |
| Primary malignant neoplasm of Rectum | 93984006 |
| Primary malignant neoplasm of Anus | 93676009 |
| Primary malignant neoplasm of Liver | 95214007 |
| Primary malignant neoplasm of Gallbladder | 372139008 |
| Primary malignant neoplasm of Extrahepatic Bile Duct | 446189008 |
| Primary malignant neoplasm of Common Bile Duct | 93763008 |
| Primary malignant neoplasm of Pancreas | 372003004 |
| Primary malignant neoplasm of Head of Pancreas | 372119009 |
| Primary malignant neoplasm of Body of Pancreas | 93715005 |
| Primary malignant neoplasm of Tail of Pancreas | 94082003 |

**Table 2. ICD-10 Codes for Malignant Neoplasms of the GI Tract**

| **GI Cancer Type** | **ICD-10 Code(s)** |
| --- | --- |
| Malignant neoplasm of Esophagus | C15, C15.3, C15.4, C15.5, C15.8, C15.9 |
| Malignant neoplasm of Stomach | C16, C16.0–C16.6, C16.8, C16.9 |
| Malignant neoplasm of Small Intestine | C17, C17.0–C17.2, C17.8, C17.9 |
| Malignant neoplasm of Colon | C18, C18.0–C18.9 |
| Malignant neoplasm of Rectosigmoid Junction | C19 |
| Malignant neoplasm of Rectum | C20 |
| Malignant neoplasm of Anus and Anal Canal | C21, C21.0–C21.2, C21.8 |
| Malignant neoplasm of Liver & Intrahepatic Bile Ducts | C22, C22.0–C22.2, C22.4, C22.7–C22.9 |
| Malignant neoplasm of Gallbladder | C23 |
| Malignant neoplasm of Other/Unspecified Biliary Tract | C24, C24.0–C24.1, C24.8, C24.9 |
| Malignant neoplasm of Pancreas | C25, C25.0–C25.4, C25.7–C25.9 |
| Malignant neoplasm of Other/Ill-Defined Digestive Organs | C26 |
| Secondary malignant neoplasms of Digestive Organs | C78.4–C78.8 |

**Table 3. CPT and ICD-10-PCS Codes for GI Cancer Surgery Procedures**

**Proctectomy and Rectal Procedures**

| **Procedure** | **CPT Code(s)** | **ICD-10-PCS Code(s)** |
| --- | --- | --- |
| Proctectomy; complete, combined abdominoperineal, with colostomy | 45110 |  |
| Proctectomy; partial resection of rectum, transabdominal approach | 45111 |  |
| Proctectomy, combined abdominoperineal, pull-through procedure (e.g., colo-anal anastomosis) | 45112 |  |
| Proctectomy, partial, with rectal mucosectomy, ileoanal anastomosis, creation of ileal reservoir (S or J) | 45113 |  |
| Proctectomy, partial, with anastomosis; abdominal and transsacral approach | 45114 |  |
| Proctectomy, combined abdominoperineal pull-through with colonic reservoir (J-pouch), with diverting enterostomy | 45119 |  |
| Proctectomy, complete (for congenital megacolon), abdominal & perineal, with pull-through & anastomosis | 45120 |  |
| Proctectomy, partial, without anastomosis, perineal approach | 45123 |  |
| Excision of rectal tumor by proctotomy, transsacral or transcoccygeal approach | 45160 | (Rectum/Anus Excision: 0DBP0, 0DBP3, 0DBP4, etc.) |
| Excision of rectal tumor, transanal approach | 45170 |  |
| Excision of rectal tumor, transanal, partial thickness (not incl. muscularis propria) | 45171 |  |
| Excision of rectal tumor, transanal, full thickness (incl. muscularis propria) | 45172 |  |
| ICD-10-PCS: Excision/Resection/Extirpation of Rectum/Anus (multiple codes) | - | 0DBP0, 0DBP3, 0DBP4, 0DBP7, 0DBP8, 0DBQ0, 0DBQ3, 0DBQ7, 0DBQ8, etc. |

**Hepatectomy (Liver Resection)**

| **Procedure** | **CPT Code(s)** | **ICD-10-PCS Code(s)** |
| --- | --- | --- |
| Hepatectomy, partial lobectomy | 47120 |  |
| Hepatectomy, trisegmentectomy | 47122 |  |
| Hepatectomy, total left lobectomy | 47125 |  |
| Hepatectomy, total right lobectomy | 47130 |  |
| ICD-10-PCS: Liver Excision/Resection/Extirpation | - | 0FB00, 0FB03, 0FB04, 0FB10, 0FB14, 0FB20, 0FB24, 0FT00, etc. |

**Pancreatectomy (Pancreas Procedures)**

| **Procedure** | **CPT Code(s)** | **ICD-10-PCS Code(s)** |
| --- | --- | --- |
| Excision of lesion of pancreas (e.g., cyst, adenoma) | 48120 |  |
| Pancreatectomy, distal subtotal, with/without splenectomy; without pancreaticojejunostomy | 48140 |  |
| Pancreatectomy, distal subtotal, with/without splenectomy; with pancreaticojejunostomy | 48145 |  |
| Excision of ampulla of Vater | 48148 | (Ampulla of Vater Excision: 0FBC0, 0FBC3, 0FBC8) |
| Whipple-type procedure (pancreatectomy, proximal subtotal) with pancreatojejunostomy | 48150, 48153, 48154 |  |
| Pancreatectomy, total | 48155 |  |
| ICD-10-PCS: Pancreas Excision/Resection/Extirpation/Removal codes | - | 0FBC0, 0FBC8, 0FBD0, 0FBD8, 0FBG0, 0FBG4, 0FBG8, 0FTG0, etc. |

**Esophagectomy (Esophagus Procedures)**

| **Procedure** | **CPT Code(s)** | **ICD-10-PCS Code(s)** |
| --- | --- | --- |
| Excision of lesion, esophagus, with primary repair (cervical or thoracic/abdominal approach) | 43100,43101 | Excision of Esophagus: 0DB50, 0DB30, etc. |
| Total or near total esophagectomy (various approaches including transhiatal, McKeown, Ivor Lewis) | 43107,43112,43113,43117,43122,43123,43124 | Resection of Esophagus: 0DT20, 0DT24, 0DT30, etc. |
| ICD-10-PCS: Excision/Resection/Extirpation/Removal of Esophagus codes | - | 0DB1-, 0DB2-, 0DB3-, 0DB4-, 0DB5-, 0DT2-, 0DT3-, etc. |

**Gastrectomy (Stomach Procedures)**

| **Procedure** | **CPT Code(s)** | **ICD-10-PCS Code(s)** |
| --- | --- | --- |
| Excision, local; malignant tumor of stomach | 43611 | Excision of Stomach: 0DB60, 0DB64, 0DB68, etc. |
| Gastrectomy (total or partial), with various anastomoses and reconstructions (e.g., Billroth, Roux-en-Y) | 43620–43639 | Resection of Stomach: 0DT60, 0DT64, 0DT68, etc. |
| ICD-10-PCS: Extirpation and Removal of Stomach codes | - | 0DC6-, 0DP6- |

**Enterectomy (Small Intestine Procedures)**

| **Procedure** | **CPT Code(s)** | **ICD-10-PCS Code(s)** |
| --- | --- | --- |
| Enterectomy (small intestine resection, single or multiple) | 44120, 44121,44202,44203 | Excision of Small Intestine: 0DB8-, 0DB9-, 0DBA-, 0DBB-, etc. |
| Enterectomy, resection with enterostomy | 44125 | Resection of Duodenum/Jejunum/Ileum: 0DT9-, 0DTA-, 0DTB- |
| ICD-10-PCS: Extirpation codes for Duodenum, Jejunum, Ileum | - | 0DC9-, 0DCA-, 0DCB- |

**Colectomy (Large Intestine Procedures)**

| **Procedure** | **CPT Code(s)** | **ICD-10-PCS Code(s)** |
| --- | --- | --- |
| Colectomy (partial, total, with/without anastomosis, various techniques) | 44140–44147,44150–44160,44204–44213 | Excision/Resection of Large Intestine: 0DBE-, 0DBF-, 0DBG-, 0DTG-, etc. |
| Mobilization of splenic flexure (add-on) | 44139,44213 |  |
| ICD-10-PCS: Extirpation and Removal codes for Lower Intestinal Tract | - | 0DC8-, 0DCE-, 0DCH-, 0DCK-, 0DCN-, 0DPD- |

**Cholecystectomy (Gallbladder Procedures)**

| **Procedure** | **CPT Code(s)** | **ICD-10-PCS Code(s)** |
| --- | --- | --- |
| Gallbladder excision/resection (cholecystectomy) | - | Excision/Resection of Gallbladder: 0FB4-, 0FT4- |
| ICD-10-PCS: Extirpation/Removal of Gallbladder | - | 0FC4-, 0FP4- |

**Excision of Biliary Tract (Hepatic/Bile Duct Procedures)**

| **Procedure** | **CPT Code(s)** | **ICD-10-PCS Code(s)** |
| --- | --- | --- |
| Excision of bile duct tumor, extrahepatic | 47711 | Excision/Resection of Hepatic/Bile Duct: 0FB5-, 0FB6-, 0FB7-, 0FB9-, 0FT7-, 0FT8-, etc. |
| ICD-10-PCS: Extirpation/Removal of Hepatobiliary Ducts | - | 0FC5-, 0FC6-, 0FC7-, 0FC8-, 0FPB- |

**Other Procedures (Omentum, Mesentery, Peritoneum, etc.)**

| **Procedure** | **CPT Code(s)** | **ICD-10-PCS Code(s)** |
| --- | --- | --- |
| Excision of presacral or sacrococcygeal tumor | 49215 |  |
| Excision/Resection of Omentum, Mesentery, Peritoneum | - | 0DBS-, 0DBT-, 0DBU-, 0DBV-, 0DBW-, 0DTS-, 0DTU-, 0DCS-, etc. |
| Extirpation/Removal of Omentum, Mesentery, Peritoneum | - | 0DCU0, 0DCV0, 0DCW0, etc. |

*Note: Some ICD-10-PCS codes are represented by patterns (e.g., 0DBP0, 0DBP3, 0DBP4, etc.) indicating a family of codes for different approaches and techniques. “-” indicates multiple related codes or code variations not individually listed. Deprecated codes are noted parenthetically where applicable.*

**Table 4.** **Distribution of Secondary GI Malignancy Diagnoses and Corresponding Surgical Procedures (n = 50)**

| **Diagnosis Site** | **No. of Patients** | **Most Common Surgical Procedures (No. of Patients)** |
| --- | --- | --- |
| Retroperitoneum | 19 | Colectomy (n = 7), Hepatectomy (n = 4), Other (n = 8) |
| Large intestine | 8 | Colectomy (n = 7), Enterectomy (n = 2), Other (n = 2) |
| Liver | 7 | Hepatectomy (n = 5), Cholecystectomy (n = 2) |
| Respiratory and digestive organs | 10 | Colectomy (n = 4), Hepatectomy (n = 2), Other (n = 4) |
| Small intestine | 5 | Enterectomy (n = 4), Colectomy (n = 2) |
| Liver and intrahepatic bile ducts | 1 | Hepatectomy (n = 1) |

**Analytical methods of specific SDoH constructs**

***Social and Community Variables***

The assessment of social cohesion and support was conducted through a series of survey items addressing neighborhood trust, shared values, and mutual assistance, providing insights into participants' perceptions of their social environments. Social support was further quantified using the RAND MOS Social Support Survey, which evaluated emotional and instrumental support. Higher scores on this scale corresponded to stronger support networks. Social cohesion was analyzed using tertiles, with scores ranging from 1.0 to 3.0 categorized as tertile 1 (low social cohesion), 3.0 to 3.6 as tertile 2 (medium social cohesion), and 3.7 to 5.0 as tertile 3 (high social cohesion). To quantify social support, the RAND MOS Social Support Survey guidelines were applied, which recommend calculating the percentage of social support by subtracting the minimum possible score from the observed score, dividing by the range (maximum minus minimum), and multiplying by 100. This method yielded a continuous variable representing the percentage of social support, with higher percentages indicating stronger perceived support.


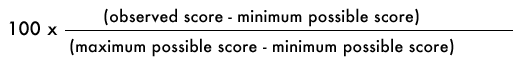


Social support was then further stratified into three tertiles, similar to the approach for social cohesion. Loneliness was categorized using the UCLA Loneliness Scale, which created four groups based on total score: low loneliness (8-13), moderate loneliness (14-19), high loneliness (20-25), and very high loneliness (26-32). Subgroup comparisons were made based on these categorizations, and statistical analysis was conducted by calculating the number of participants in each group to assess the distribution of loneliness within the cohort.

Perceived discrimination was measured through both general and healthcare-specific experiences, with frequency data facilitating comparisons across demographic subgroups. The absence of a standard scale for perceived discrimination in the All of Us data survey led to the use of a Likert scale (1-6) to assess the mean level of discrimination in the cohort, with higher mean scores indicating higher levels of perceived discrimination. Perceived stress was measured using the Cohen Perceived Stress Scale (PSS-10), where scores were interpreted as follows: low stress (0-13), moderate stress (14-26), and high stress (27-40). For perceived spiritual experience, the Daily Spiritual Experience Scale (DSES) was employed. However, given the absence of a reference group, comparisons based on average scores could not be made in this study, and no universal thresholds for high, medium, or low scores are applicable according to the DSES guidelines.

***Neighborhood and Built Environment Variables***

Neighborhood conditions were assessed using a comprehensive approach that captured both physical and social disorder, including the prevalence of graffiti, noise, crime, and drug use, as well as neighborhood walkability and access to public amenities. These environmental variables were measured through a series of items focused on physical and social disorder, such as perceived crime, noise, and accessibility to recreational facilities. For physical and social disorder, the Ross-Mirowsky Perceived Neighborhood Disorder Scale was used, with participant responses scored on a Likert scale ranging from 1 to 4. A mean score was calculated for each participant, and these individual means were then averaged to provide a composite measure of neighborhood disorder. Similarly, neighborhood crime, walkability, and residential density were assessed using a modified version of the Physical Activity Neighborhood Environment Scale (PANES). Since PANES lacks a predefined scoring system, the same analysis method was applied, calculating the mean for each participant and then averaging across all participants. Higher mean scores for walkability indicated neighborhoods with better walkability, whereas higher mean scores for crime reflected higher perceived levels of crime.

***Economic Stability***

Economic stability was evaluated through indicators of food and housing insecurity, as well as housing quality. Food insecurity was assessed using the Hunger Vital Sign™, a two-question survey, where a response of "sometimes true" or "often true" to either question was considered indicative of food insecurity. Housing insecurity was measured using the Health Begins Social Screening survey, with participants scoring positive for housing instability if they had moved more than twice within the past year. Housing quality was assessed using the Health-Related Social Needs (HRSN) survey, developed by the Center for Medicare and Medicaid Services (CMS). A positive HRSN score indicated poor housing quality, which was identified by any response other than “none of the above.” Statistical analysis was conducted to calculate the proportion of participants screening positive for food insecurity, housing instability, and poor housing quality.

***Lifestyle and Health Behaviors***

The Lifestyle survey provided additional data on health behaviors, including physical activity, diet, tobacco and alcohol use, and sleep patterns. The Overall Health survey captured self-reported health status, physical and mental health symptoms, and diagnoses. The Healthcare Access and Utilization survey provided insights into healthcare service utilization, usual sources of care, and barriers to access such as insurance limitations and affordability. Alcohol consumption was assessed using the AUDIT-C scale, with a score of 4 or more for men and 3 or more for women indicating at-risk drinking. Participants were grouped into four categories based on their total AUDIT-C score: low risk, moderate risk, high risk, and severe high risk. The mean AUDIT-C scores were then used to assess the prevalence of potentially hazardous drinking. Illicit drug use was not analyzed in depth due to low response rates.

Health-related quality of life was assessed using the PROMIS global health scales for physical and mental health, where scores below the population mean indicated impairments. These impairments were further categorized into mild, moderate, and severe levels, allowing for a stratified analysis of physical and mental health challenges.

***Statistical Analysis***

Descriptive statistics, including means, standard deviations, and tertile distributions, were calculated for each domain of social determinants of health (SDoH). Comparative analyses across demographic groups were conducted where appropriate to identify patterns and disparities in health outcomes. These analyses aimed to uncover relationships between SDoH factors and health outcomes within the cohort, providing insights into potential drivers of health inequities.
